# Recommendations of an Independent Expert Committee for the Development of Quebec’s First Government Policy on Primary Care

**DOI:** 10.1177/08404704251397322

**Published:** 2026-01-21

**Authors:** Mylaine Breton, Elise Boulanger, Catherine Lamoureux-Lamarche, Marie-Dominique Beaulieu, Karina Prévost, Sophie Boies, Catherine Bouffard-Dumais, Antoine Groulx

**Affiliations:** 1 Department of Community Health Services, 198734 Université de Sherbrooke, Longueuil, Quebec, Canada; 2 Indigo Clinic, Montréal, Quebec, Canada; 3 Department of Family and Emergency Medicine, 12368 Université de Montréal, Montréal, Quebec, Canada; 4 Unité de Soutien SSA Quebec, Longueuil, Quebec, Canada; 5 Department of Family and Emergency Medicine, 12369 Université Laval, Quebec City, Quebec, Canada

## Abstract

In January 2025, the Ministry of Health and Social Services commissioned an independent expert panel to make recommendations to guide the first government policy addressing the primary care crisis in Quebec. Conducted over a period of 4 months, this work combines a targeted literature review, 59 consultations with more than 200 stakeholders, and a provincial forum to develop recommendations grounded in evidence and local realities. Findings from the consultations revealed a fragmented, hospital-centred system characterized by inequitable access and insufficient continuity. The expert committee formulated six coherent and locally adaptable key recommendations aligned with international best practices. This provides a pragmatic and comprehensive roadmap to strengthen primary care in Quebec through interdisciplinary teams, territorial governance, protected and dedicated funding, data infrastructure, and user involvement. A concrete action plan is essential to achieve the proposed vision, which will require time, consistency, and structured planning.

## Introduction

Primary Care (PC) is the foundation of healthcare systems, delivering essential services that meet 80% of health needs throughout an individual’s lifetime.^1^ Decades of research have shown that health systems with strong PC have better health outcomes and greater equity as well as lower costs.^2,3^ PC refers to direct care and services to individuals.^4,5^ It brings together a range of professionals, covers a wide variety of activities (prevention, treatment, and follow-up),^6,7^ and is deployed in local environments and communities.

In Canada, 22% of the population does not have access to a regular source of PC,^8^ with the province of Quebec having one of the highest proportions at 31%.^9^ Furthermore, among those who do have a regular source of PC, many experience issues gaining timely access.^10^ Most provinces have undertaken reforms to address the PC crisis, including the Primary Care Act legislation that is part of the Ontario government’s Primary Care Action plan^11,12^ and British Columbia’s reform of the physician payment system.^13^ Quebec has chosen to draft its first government policy on PC to establish a strategic vision to guide planned transformations. It was in this context that Quebec’s Minister of Health and Social Services commissioned an independent panel of experts to produce recommendations based on scientific and experiential knowledge to inform the drafting of a government policy on PC. The aim of this article is to summarize the independent expert panel’s report^14^ as well as offer a rich discussion based on the Quebec context, work conducted in other jurisdictions, and next steps for implementing this first policy on PC in Quebec. This approach could serve as a guide for other jurisdictions on the development of public health policies.

## Methods

The methodology for this mandate is based on three sequential and complementary components combining scientific and experiential knowledge, carried out over a 4-month period.

### Component 1

A review of relevant scientific literature synthesis and publications by national and international organizations on the characteristics of effective PC systems was conducted. The review included a jurisdictional analysis of six countries (United Kingdom, Sweden, Netherlands, Norway, France, and Canada) with similar context and three provinces (Ontario, Alberta, and Quebec), based on the Lamarche framework.^15^

Given the time constraints to complete the mandate, we performed targeted reviews of effective PC characteristics with limited evidence, such as continuity of care, formal attachment to a healthcare provider or care setting, PC teams, and territorial governance. These reviews were guided by the expertise of our team over the past 20 years and accumulated experience in the context of other mandates. Additional searches were carried out in relevant databases (i.e., PubMed, Medline, and Google Scholar) and on web sites from governmental, provincial, national, and international organizations to identify key articles and documents. Finally, we conducted three consultations with 25 researchers, inviting them to share relevant articles and documents to be included in the review.

### Component 2

Fifty-nine consultations were conducted through semi-structured individual or group interviews with over 210 participants from various regions of Quebec, representing key stakeholders in PC—including user-partners, healthcare professionals, researchers, professional associations and regulatory bodies, health system managers, government representatives, and community organizations. The interviews were not recorded, but notes were taken and summarized. The objective of these consultations was to examine how the findings from the literature synthesis could be applied to the Quebec context and to gather experiential knowledge from the field.

### Component 3

An in-person forum consultation and reflection on proposed recommendations was held on March 20, 2025, and attended by 135 individuals, the majority of whom had participated in the interviews. The aim of the forum was to enrich the preliminary recommendations drawn up by the expert committee following the literature review and consultations, not to reach a consensus among all participants.

The expert committee then met for 2 days to draft the final recommendations based on the values and orientations of Quebec’s health and social services system, aligned with current provincial policies and initiatives, and guided by the quintuple aim.^16^

In this article, we present the main findings from the literature review (component 1) and stakeholder consultations (components 2 and 3). We then highlight areas of divergence between the scientific evidence and stakeholder perspectives, as well as among stakeholders themselves. The article concludes with a set of recommendations formulated by experts in the specific context of Quebec. Ethical approval was received from the Research Ethics Board of the Centre intégré universitaire de santé et de services sociaux de l'Estrie - Centre hospitalier universitaire de Sherbrooke (#2025-5839).

## Results

### Synthesis of the Evidence on Characteristics of Effective PC Systems

The synthesis of evidence showed that there is no single model of efficient PC; rather, the success of transformations depends on coherent configurations between the different components of the system and adaptations to the cultural and socio-political context.^15^ However, an international consensus is emerging regarding the essential characteristics of high-performing systems^15^: (1) explicit policies rooted in societal values and needs (vision); (2) financing and remuneration mechanisms aligned with health objectives (resources); (3) local, regional, and national governance structures in partnership with communities (structure); (4) formal enrolment to reinforce continuity and accountability (structure) of interprofessional teams; and, finally, (5) close coordination with other services in the territory (practices). These characteristics are underpinned by cross-cutting factors: user involvement, information technology, data infrastructure, continuous performance evaluation, support for quality improvement, leadership development, and research capacity.^15^

Access—defined as the ability to obtain necessary healthcare services in a timely manner—is a cornerstone of strong and effective PC, alongside relational continuity.^17^ However, these two fundamental functions are often in tension.^18^ Relational and team continuity improve diagnostic accuracy, appropriateness of treatment plans, and health outcomes, including reduced mortality.^19,20^ The data demonstrate that the higher the continuity, the more significant the benefits, notably in terms of reductions in hospitalizations, emergency room visits, and overall system costs.^21^ Furthermore, access and continuity are interdependent.^22,23^ Public policy must support comprehensive continuity measures to avoid fragmentation of care and move beyond approaches focused solely on rapid access.

**Figure 1:**
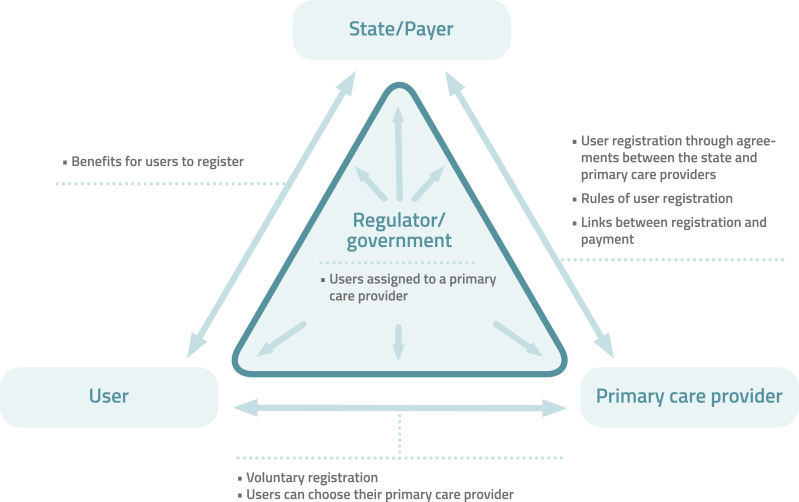
Adaptation of Registration Triad from Marchildon et al. (2021)

Formal attachment (registration) is first and foremost a public policy instrument designed to facilitate continuity of care and establish a mechanism of accountability between users, professionals or clinics, and the State.^24^ Figure 1 illustrates this triad of responsibilities within the public policy tool of registration. It formalizes the relationship of shared responsibility for a population, thereby supporting the organization and financing of services. However, registration in itself does not guarantee continuity. To be effective, it must be part of an overall framework aligning a coherent set of measures, including appropriate remuneration arrangements, clear contracts with professionals, an organization of services centred on the needs and expectations of the population, as well as effective local governance and robust accountability mechanisms.^24^ Although the literature examining the impact of patient registration on the effectiveness of PC remains limited, existing studies on the loss of formal attachment to a provider suggest that such disruptions may have significant consequences for patients’ care experiences, healthcare utilization patterns, and system-level costs.^25^

The optimal composition of PC teams varies according to context but generally includes a core of family physicians or nurse practitioners in PC, nurses, social workers, pharmacists, and administrative staff.^26,27^ Team effectiveness depends on a clear division of roles, the broadening of professional practices, and the integration of users as full team members.^28-30^ Models with a high number of professionals per doctor enable a greater number of patients to be cared for.^31^

Territorial governance refers to the organization, management, and coordination of healthcare services at the local level to ensure equitable access to quality care for the population.^32^ The level of territorial division for the organization of PC should ensure geographical proximity and accessibility to services, meet the population’s needs, and allow for efficient management of resources. Local leadership, in-depth knowledge of the context, and strong relationships with key actors in communities are widely recognized as necessary to manage the realities of population-based PC coverage, supporting the argument for a smaller level of territorial governance.^33^

Finally, PC policy monitoring indicators must focus on value creation and relate to outcomes that are important to the population.^34^ The quintuple aim may serve as a framework to develop a set of such indicators.^16^

### Findings from the Consultations and the Forum

The consultations enabled the collection of experiential knowledge from a broad spectrum of stakeholders, including patient partners. They allowed for contextualization of the findings from the literature within the specific realities of the Quebec healthcare system and supported the identification of innovative strategies to address current challenges. The main finding from the consultations was a strong consensus to anchor PC in a genuine social project. At the heart of this vision, health is perceived as a collective good that is built everywhere and upstream of illness, through active participation of the population. This calls for concrete action to promote the psychological, mental, physical, and social well-being of everyone.

The analysis revealed a portrait of a fragmented PC system that remains predominantly hospital- and illness-centred. Collaboration is hindered by structural silos, misunderstanding of roles, and administrative complexity. The growing disengagement of healthcare professionals is driven by insufficient recognition and burdensome working conditions. Access to care remains inequitable and difficult, particularly for vulnerable populations, while promising innovations struggle to move beyond the experimental stage due to a lack of long-term support. Current performance indicators, focused mainly on volume, limit the ability to assess quality of care and support continuous improvement. Proposed solutions converge on the need for dedicated and decentralized funding mechanisms, strengthened interprofessional team-based care, the development of a value-oriented data culture, enhanced citizen engagement, flexible models of workforce participation, and localized governance structures that are better aligned with community needs.

### Divergences between Evidence from the Literature and the Consultations

Cross-analysis of consultations and evidence from the literature is essential to identify the levers of transformation on which to build and to anticipate areas of tension to be addressed in implementation.

Several significant divergences deserve particular attention. The concept of registration or formal attachment to a provider was often misunderstood or questioned in consultations. Several of those consulted saw this as an unworkable mechanism in the current context, notably because of the lack of human resources and the population’s current experience of formal attachment with a family physician unable to provide real access in a timely manner.

The issue of territorial governance remains contested, with no consensus on the optimal level of granularity—whether at the scale of local health networks, local community service centres, or other territorial units—or on the specific levers of action available to local actors. Although the importance of social determinants of health is widely acknowledged, intersectoral collaboration with key community sectors—such as education, housing, and municipal services—remains weak and insufficiently institutionalized. Consultations revealed a lack of structured mechanisms and a heavy reliance on isolated local initiatives, limiting the populational scope of PC interventions.

The preponderance of medical leadership is being questioned, and the terms of co-management of care remain unclear for many professionals. In the predominant PC model in Quebec, the Family Medicine Group (FMG), allied healthcare professionals are hired by the regional public health institution, while family physicians remain autonomous professionals in privately owned facilities. The dual affiliation of professionals was perceived as a hindrance to collaboration. This model of financing and integrating non-physician professionals with an employment contract outside the clinic was not observed in the other jurisdictions analyzed. Several people consulted described tensions related to the coordination of schedules, organizational culture, and accountability, with no clearly identified levers to resolve them.

Information technologies, although identified as levers for integrating high-performing systems, are often poorly adapted to actual workflows, impose an administrative burden, and are rarely developed with significant input from professionals and the public. Consultations reported little support for technological tools to foster care coordination, information sharing, and collaboration.

### Six Recommendations for a Government Policy on PC in Quebec

**Figure 2.**
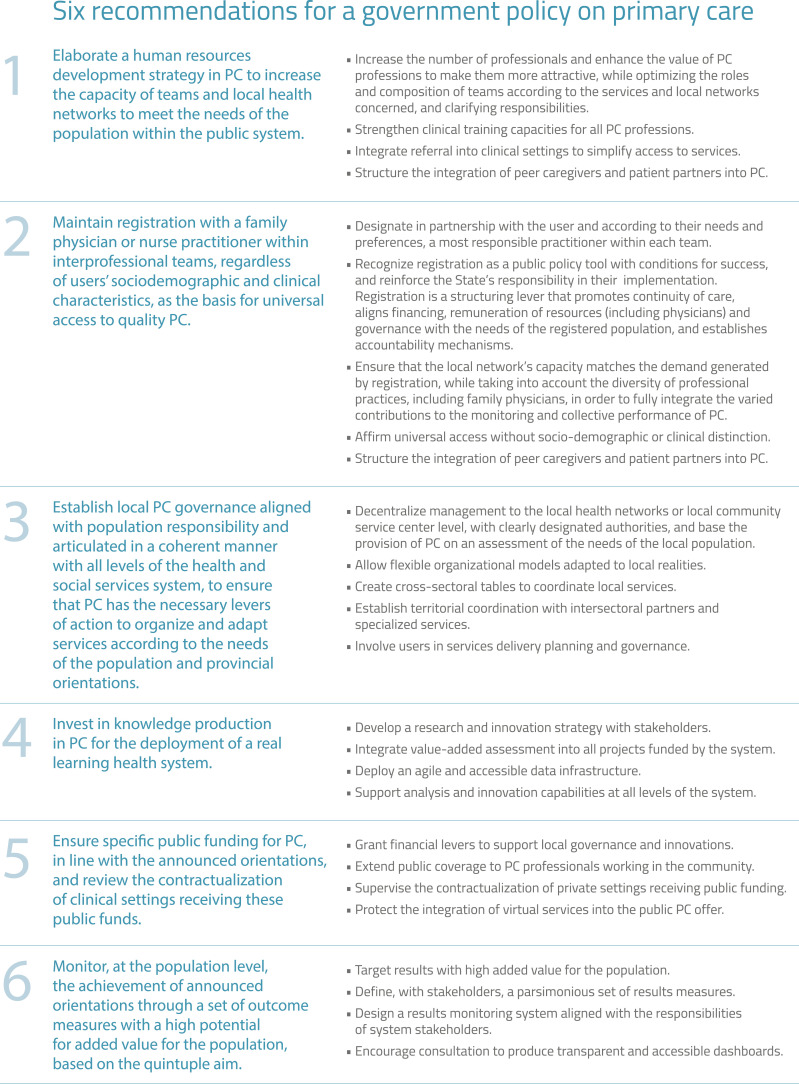
Six Recommendations for a Government Policy on PC

Based on the analysis of the scientific literature, consultations, and the forum, our expert committee identified six recommendations for a government policy on PC, presented in Figure 2. Analysis of divergences helped to identify the priority areas to be addressed to ensure buy-in, equity, and the feasibility of the changes envisaged in future public policy. Additionally, several of the recommendations for transforming PC are consistent with international best practices.

## Discussion

At the federal level, the Canada Health Act, adopted in 1984, establishes the overarching framework ensuring that PC services across all provinces and territories are publicly funded and accessible without direct charges to patients.^35^ Until recently, however, no province had a dedicated PC policy. Innovative approaches have emerged in certain jurisdictions, such as Alberta’s Integrated Health Neighbourhood of the Future,^36^ which seeks to strengthen PC through community-based, interdisciplinary models of service delivery, and British Colombia’s reform of the physician payment system.^13^ In 2025, Ontario became the first province to enact a Primary Care Act (2025),^37^ marking a significant milestone in formalizing government commitment to PC. In January 2025, in Quebec, the Ministry of Health and Social Services announced the development of the first government policy on PC and mandated an independent expert committee to formulate evidence-informed recommendations to guide this initiative. The recommendations presented in this article are adapted to the specific context of Quebec and based on a rigorous approach that emerged from a literature review and multi-stakeholder consultations.

The vision underpinning the recommendations made by the expert committee is built on Quebec’s existing achievements in PC. First, the province has developed a network of FMGs, which represent the main model of PC clinics in Quebec and is based on an interdisciplinary model that extends to 65% of the Quebec population.^38^ This is the highest population coverage for an interdisciplinary model in Canada.^39^ While the FMG model is advantageous, there remain significant challenges to timely access for registered patients in Quebec.^8,10^ Ensuring an appropriate balance between the number of attached patients and the availability of healthcare professionals is essential to avoid further compromising timely access to care. Over-registration of patients beyond a clinic’s capacity may undermine timely access and lead to increased wait times and reduced continuity of care.^40,41^ In addition, recent policy and regulation adjustments have broadened the scope of practice for multiple healthcare professions, such as nurse practitioners and pharmacists,^42,43^ enabling greater mobilization of diverse professional capacities to enhance access and delivery of PC services. Patient registration capacity must be considered through an interdisciplinary approach, where each professional contributes according to their role. A team’s overall capacity to provide care increases with the number of professionals per family physician or nurse practitioner. International data show that models based primarily on physicians result in a capacity to provide care for approximately 900 patients per Full-Time Equivalent (FTE) physician, whereas interdisciplinary teams with a 3:1 ratio can reach around 2,000 patients, with capacity increasing with higher ratios.^31^ At the same time, a cultural shift is needed to enhance patients’ understanding of the collaborative approach and the role of each professional to optimize quality of care.

Another key element emerging from the literature review and stakeholder consultations was the notion of *local* territorial governance. This concept is closely tied to the principle of population-based responsibility, which seeks to maintain and improve the health and well-being of a geographically defined population.^44^ One of our six recommendations is to establish a formal structure for local territorial governance, which draws on a long-standing tradition of territorially organized PC services in Quebec, rooted in the Community-based Local Health Centre (CLSC) model. Although this model has been partially eroded by successive reforms,^45^ it remains a foundational element of the province’s approach to PC. Within Quebec’s current highly centralized system, PC falls under the purview of several distinct institutional directorates that often operate in silos.^46^ The absence of a dedicated PC governance structure at the local level—combined with the limited authority of existing coordination mechanisms—constrains the system’s ability to support concerted, population-based action.

We can draw inspiration from other countries that have organized and delivered PC services according to a territorial and community-based approach. For example, England provides one of the most advanced examples of territorial governance, having established Primary Care Networks (PCNs) in 2019 to promote care integration within local communities. These networks consist of groups of general practitioners working collaboratively with various local stakeholders, including community-based organizations, and typically serve populations of 30,000 to 50,000 residents.^31,47^ The model is financed through a shared-savings contract that reinvests efficiency gains into the territorial integration of services.^31,47^ In Scandinavian countries, by contrast, municipalities are responsible for both the financing and organization of PC services, illustrating a deeply decentralized governance model that embeds accountability within the local political and administrative context.^31,48,49^

Of the themes that elicited divergence during our consultations, the concept of patient registration emerged as particularly contentious, reflecting varying interpretations of the underlying purpose, implementation modalities, and anticipated outcomes. In Quebec, patient registration with a family physician or nurse practitioner has yet to harness the full array of success factors typically associated with this mechanism. Consistent with this observation, Marchildon et al. concluded that few jurisdictions have benefited from the lever associated with patient registration.^24^ Formal attachment to a regular source of PC as a public policy mechanism requires a set of consistent measures in terms of professional remuneration, contractualization with providers, organization of services, governance, and accountability.^50^

The first governmental PC policy in Quebec is expected to be launched in the fall of 2025. Its success will depend on the development of a structured transition plan to guide and support the progressive implementation of the policy across the province. In Ontario, the government has directly put in place a PC action plan that includes staggered implementation with attachment milestones to ensure full population attachment to a family doctor or PC nurse practitioner by 2029.^51^ One of the three strategies of the plan is to develop 305 PC teams including family doctors, nurse practitioners, and other allied health professionals. It appears essential for Quebec to adopt a similarly pragmatic approach, establishing realistic and context-sensitive targets that reflect existing organizational structures and system capacities.

To ensure relevance and utility in informing Quebec’s first government policy on PC, the mandate of the independent experts had to be completed within a constrained timeframe of 4 months. This posed a considerable challenge for the team, which conducted the majority of the 60 consultations with various stakeholder groups within a 5-week period. Nevertheless, despite these time constraints, all key stakeholders relevant to PC were engaged in the consultation process. The resulting recommendations have been drawn up for the Quebec context and can be adapted to other contexts.

## Conclusion

This article proposes a structured approach to formulate recommendations intended to serve as the foundation for a government policy. The recommendations that emerged from this process outline the core elements of a strong, coherent, and mobilized PC system for the Quebec population. The success of this transformation will rely on thoughtful and consistent implementation. Both wall-to-wall approaches and selective adoption of recommendations (“cherry-picking”) should be avoided, as they risk undermining the overall coherence and impact of the reform. A concrete action plan, co-developed with stakeholders, is essential to achieve the proposed vision, which will require sustained effort, consistency, and carefully structured planning.

## Data Availability

The consultations and discussions during the Forum were not recorded. Summaries were produced by the research team afterwards.*
